# Natural graft tissues and synthetic biomaterials for periodontal and alveolar bone reconstructive applications: a review

**DOI:** 10.1186/s40824-017-0095-5

**Published:** 2017-06-05

**Authors:** Zeeshan Sheikh, Nader Hamdan, Yuichi Ikeda, Marc Grynpas, Bernhard Ganss, Michael Glogauer

**Affiliations:** 1 Matrix Dynamics Group, Faculty of Dentistry, University of Toronto, Room 221, 150 College Street, Toronto, ON M5S 3E2 Canada; 2 Lunenfeld-Tanenbaum Research Institute, Mt. Sinai Hospital, 25 Orde St, Toronto, ON M5T 3H7 Canada; 3 Department of Dental Clinical Sciences, Faculty of Dentistry, Dalhousie University, 5981 University Avenue, PO Box 15000, Halifax, Nova Scotia B3H 4R2 Canada; 4 Department of Periodontology, Graduate School of Medical and Dental Sciences, Tokyo Medical and Dental University, 1-5-45 Yushima Bunkyo-ku, Tokyo, 113-5810 Japan

## Abstract

Periodontal disease is categorized by the destruction of periodontal tissues. Over the years, there have been several clinical techniques and material options that been investigated for periodontal defect repair/regeneration. The development of improved biomaterials for periodontal tissue engineering has significantly improved the available treatment options and their clinical results. Bone replacement graft materials, barrier membranes, various growth factors and combination of these have been used. The available bone tissue replacement materials commonly used include autografts, allografts, xenografts and alloplasts. These graft materials mostly function as osteogenic, osteoinductive and/or osteoconductive scaffolds. Polymers (natural and synthetic) are more widely used as a barrier material in guided tissue regeneration (GTR) and guided bone regeneration (GBR) applications. They work on the principle of epithelial cell exclusion to allow periodontal ligament and alveolar bone cells to repopulate the defect before the normally faster epithelial cells. However, in an attempt to overcome complications related to the epithelial down-growth and/or collapse of the non-rigid barrier membrane and to maintain space, clinicians commonly use a combination of membranes with hard tissue grafts. This article aims to review various available natural tissues and biomaterial based bone replacement graft and membrane options used in periodontal regeneration applications.

## Background

It has been estimated that the global economic cost incurred due to dental diseases amounted to $442 Billion in 2010, of which $298 Billion can be attributed to direct treatment costs and $144 Billion to indirect costs in terms of productivity losses due to periodontal disease, caries and tooth loss [1]. Chronic periodontitis is a disease that affects approximately half of the adult population in the United States [2], of those, it is estimated that 2 to 6 million people could require professional treatment. Since the average cost for full mouth periodontal surgery is about $4000 to $5000, and if 300,000 people only actually received treatment, the projected cost could be more than one billion dollars. This would be an overwhelming liability for insurance companies and health care plans to cover. This out-of-pocket cost to the individual would contribute in discouraging some individuals from seeking treatment [3]. The chronic untreated loss of periodontal tissues: gingiva, alveolar bone, periodontal ligament and cementum, ultimately results in tooth loss leading to functional and aesthetic repercussions. Various treatment modalities (surgical and non-surgical) have been investigated to try repair/regenerate periodontal tissues damaged or lost due to disease. In an attempt to achieve periodontal regeneration, soft and hard tissue replacement grafts, guided tissue/bone regeneration (GTR/GBR), root surface biomodifications, and delivery of growth factors have been developed [4]. Four major hard tissue replacement graft materials are commonly used for periodontal regenerative applications. These are the autogenous or autografts, allografts, xenografts and alloplasts. Autografts are graft materials obtained from the same individual and have been historically thought to be the “gold standard” [5]. However, there are concerns about donor site morbidity [6], the volume of bone acquired is usually limited, and the replacement rate of those autografts may be unpredictable [7]. Allografts are derived from a donor of the same species, which may be a fresh/frozen, freeze-dried bone or demineralized freeze-dried bone [8]. These allografts can act not only as osteoconductive scaffolds, but may also have some osteoinductive potential, due to the presence of proteins such as bone morphogenetic proteins (BMP) [9]. Xenografts are obtained from another species and are widely used in clinical periodontal regenerative applications. Alloplastic materials include ceramics and polymers and are either natural or synthetic. They have no risk for cross infection/disease transmission, which might be a possibility with the use of allografts and xenografts [10]. To prevent the down-growth of the epithelial cells along the tooth-root surface and into the periodontal defect space, various barrier membranes have been developed and investigated [11]. Similar to the hard tissue replacement graft materials, these membranes can be manufactured using natural or synthetic materials [12]. In this review, we will focus on the natural tissues and synthetic biomaterials used in periodontal regeneration; discuss their properties and applications and also the future prospects.

## Natural tissues and synthetic materials as bone replacement grafts

There are various hard tissue replacement materials available and divided into natural transplants (autografts, allografts and xenografts) and synthetic materials (alloplasts) (Tables 1 and 2). These materials are used because they possess osteogenic, osteoinductive and/or osteoconductive properties [13]. These grafts should ideally be biocompatible, easily molded and/or carved, integrate well with the native bone and have adequate mechanical properties [14]. Hard tissue substitute graft materials that have the ability to be resorbed, undergo a replacement process during which they are partially or completely resorbed by macrophages/ osteoclasts before native bone is deposited by osteoblasts [15, 16]. These grafts should ideally be biocompatible, easily molded and/or carved, integrate well with the native bone, have adequate mechanical properties with an ideal replacement rate, and be predictable with a good level of patient acceptance. This section discusses the various graft tissues and biomaterial alternatives used for alveolar bone grafting and periodontal defect fill applications.

**Table 1:** Commonly used natural tissues and biomaterial graft option types for periodontal hard tissue regenerative applications classified according to source

Bone replacement graft materials	
Human bone graft tissues	
(a) Autografts (cancellous and/ or cortical)	
-Extra-oral	
-Intra-oral	
(b) Allografts (cancellous and/ or cortical)	
-Fresh and/or frozen bone	
-Freeze dried bone allograft (FDBA)	
-Demineralized freeze dried bone allograft (DFDBA)	
Non- human source materials	
(a) Xenografts	
-Bovine Hydroxyapatite	
-Porcine bone	
-Equine bone	
-Coralline calcium carbonate	
Synthetic materials (Alloplasts)	
(a) Bioactive glasses	
(b) Calcium phosphates	
-Hydroxyapatite	
-Tricalcium phosphate	
-Other calcium phosphates (Brushite,	
monetite, calcium polyphosphates/CPP)	
(c) Calcium Sulphate

**Table 2:** Examples of commercially available bone grafts for periodontal reconstructive applications

Brand name	Generic name/composition	Company	Source	Category
Puros®	Mineralized bone allograft	Zimmer Biomet	Human bone	Allograft
Raptos®	Mineralized/ demineralized bone allograft	Citagenix	Human bone	Allograft
Grafton® (DBM)	Demineralized Bone Matrix	BioHorizons	Human bone	Allograft
DBX® Putty (DBM)	Demineralized Bone Matrix	DENTSPLY	Human bone	Allograft
MTF® - FDBA	Freeze Dried Bone Allograft	Musculoskeletal Transplant Foundation	Human bone	Allograft
MTF® - DFDBA	Demineralized Freeze Dried Bone Allograft	Musculoskeletal Transplant Foundation	Human bone	Allograft
Gen-Os®	Anorganic Porcine Bone Mineral	Tecnoss Dental	Porcine bone	Xenograft
Bio-Oss®	Deproteinized Bovine Bone Mineral	Geistlich	Bovine bone	Xenograft
Osteograf/ N®	Anorganic Bovine Bone Mineral	Dentsply	Bovine bone	Xenograft
PepGen P-15®	Anorganic Bovine Bone Mineral with a synthetic biomimetic of the 15 amino acid sequence of Type-I collagen	Dentsply	Bovine bone/tissue engineering	Xenograft/synthetic
Biocoral®	Corraline Calcium Carbonate	Inoteb	marine corals	Xenograft
Interpore 200®	Porous Hydroxyapatite	Interpore International	marine corals	Xenograft
PerioGlas®	Bioactive Glass	NovaBone	Synthetic	Alloplast
Guidor easy-graft®	In situ hardening beta-tricalcium phosphate (β-TCP) granules coated with poly(lactic-co-glycolic acid) (PLGA)	Sunstar	Synthetic	Alloplast
Vitoss®	β-TCP	Stryker	Synthetic	Alloplast
Eurobone®	Dicalcium phosphate dihydrate (Brushite) - DCPD	Kasios	Synthetic	Alloplast

### Autografts

Autografts are harvested from a donor site in the same individual and transplanted to another site. Autografts are a source of the most osteogenic organic material for grafting, however, donor site morbidity, and limited graft volume that can be obtained are disadvantages [6, 17]. Autografts used in periodontal regeneration may be of extraoral or intraoral origin. Intraoral autograft harvest sites are the spina nasalis, the tuberosity and crista zygomatico-alveolaris from the maxilla, the ramus, retromolar region and the symphysis region in the mandible, as well as bony exostoses and bone harvested from different sites utilizing bone scrapers [18]. Mandibular autografts are commonly used as bone chips, blocks and milled particles [19, 20]. Autografts obtained from extraoral sites such as the iliac crest provide osteoinductive, osteoconductive and osteogeneic potential [21]. The calvaria is another extraoral site that can be used to potentially obtain bone tissue for surgical applications [22, 23]. However, there is less morbidity associated with intraoral donor sites and that is the reason they are preferred [19].

**Fig. 1:**
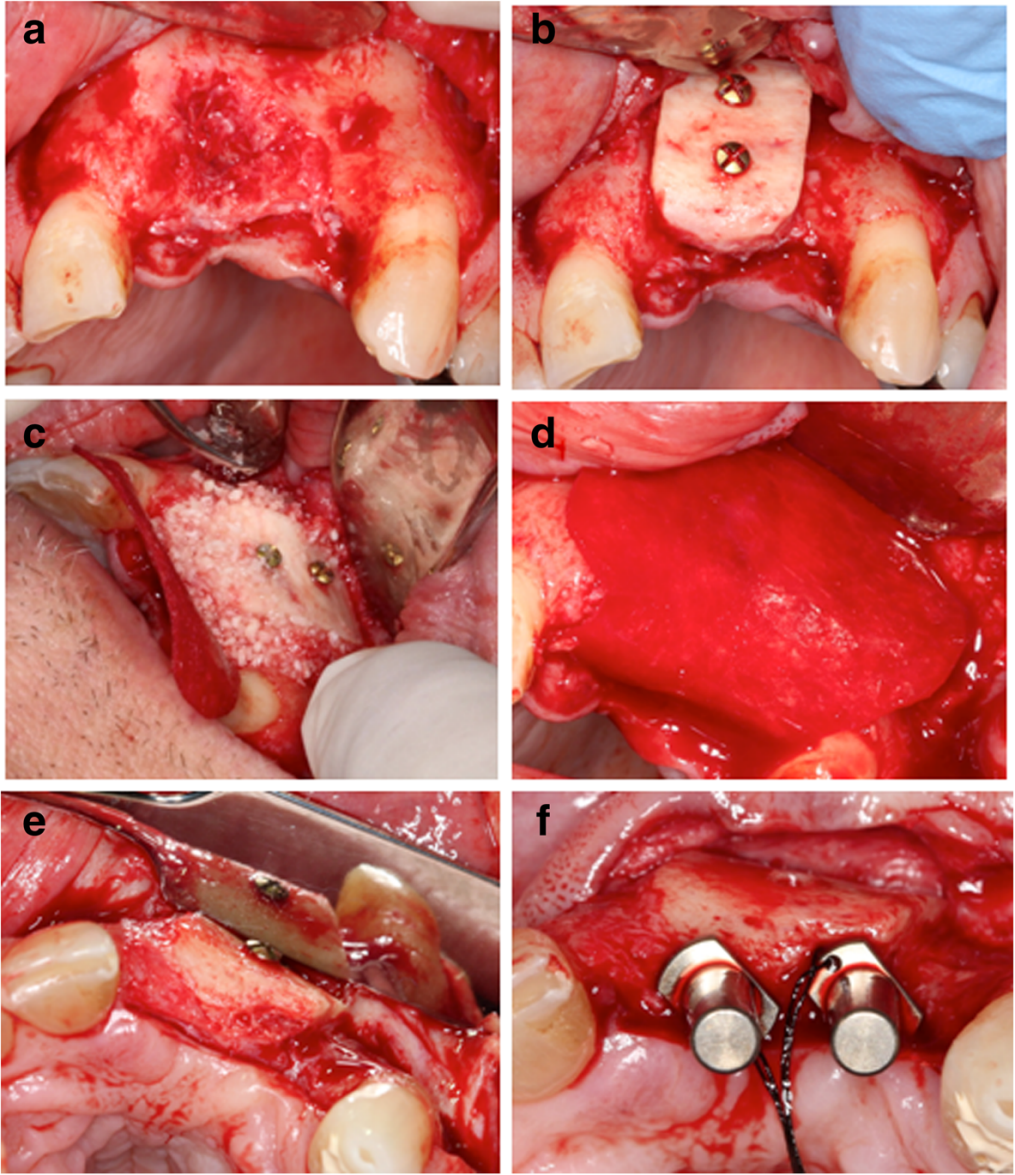
Clinical photographs showing autogenous block grafting. **a** Advanced vertical and horizontal bone loss. **b** Autogenous block graft fixed with screws. **c** FDBA particles added to fill any remaining gaps. **d** Porcine degrdable collagen membrane (Bio-Gide) used to contain and cover bone grafts. **e**. Six month results showing successful bone augmentation. **f** Dental implants successfully placed into augmented bone. (Courtesy of Dr. Aditya Patel, periodontist, Halifax, NS, Canada)

The common extraoral harvest site that provides large amounts of autologous cortical-cancellous bone is the iliac crest [24]. Cortical autografts have high initial strength which after about 6 months of implantation is about 50% weaker than the physiologically normal bone tissue [25]. Conversely, cancellous bone autografts are initially weaker because of their porous structure and gain strength over time [13]. The cancellous autografts revascularize earlier than the cortical grafts around the fifth day after implantation due to their spongy architecture [13]. Vertical and horizontal alveolar ridge augmentation using particulate autografts with GBR has been shown to be successful for placing dental implants [26, 27], (Fig. 1). However, block grafts outperform particulate grafts with regards to revascularization, bone-to-implant contact and bone remodelling [26].

### Allografts

Allografts are tissues taken from genetically non-identical members of the same species, i.e. from another human. They are available in large amounts for use and do not have the traditional shortcomings associated with autografts. Cancellous and cortical allografts of various particle sizes are regularly used for bone regeneration procedures with minimal risk of disease transmission due to the screening and virucidal tissue processing methods [28–30]. However, the possibility of tissue contamination and disease transmission with new unidentified pathogens poses some risk as these may not be eliminated through current methods of donor screening and tissue processing. Although to our knowledge no cases have been documented of prion disease from bone allografts, the concern is valid [31]. Additional factors should be taken into consideration such as human error, persistent antibody-negative carriers and immunovariant strains [32, 33]. Also, cases of infection and disease transmission may go unreported [34].

Allografts are available for periodontal applications as cortical wedges, cortical chips, cortical granules and cancellous powdered prepared as frozen, freeze-dried, mineralized and demineralized bone [35].

#### Fresh-frozen bone allografts (FFB)

Fresh frozen cancellous bone provides the highest osteoconductive and osteoinductive potential among all allograft materials available for use [36, 37]. However, due to the risk of disease transmission, fresh-frozen allografts are not used anymore. In the past, atrophic maxillary ridges grafted with human block allografts of tibia and fresh-frozen chips showed development of mature and compact osseous tissue surrounded by marrow spaces [38, 39].

#### Freeze-dried bone allografts (FDBA)

**Fig. 2:**
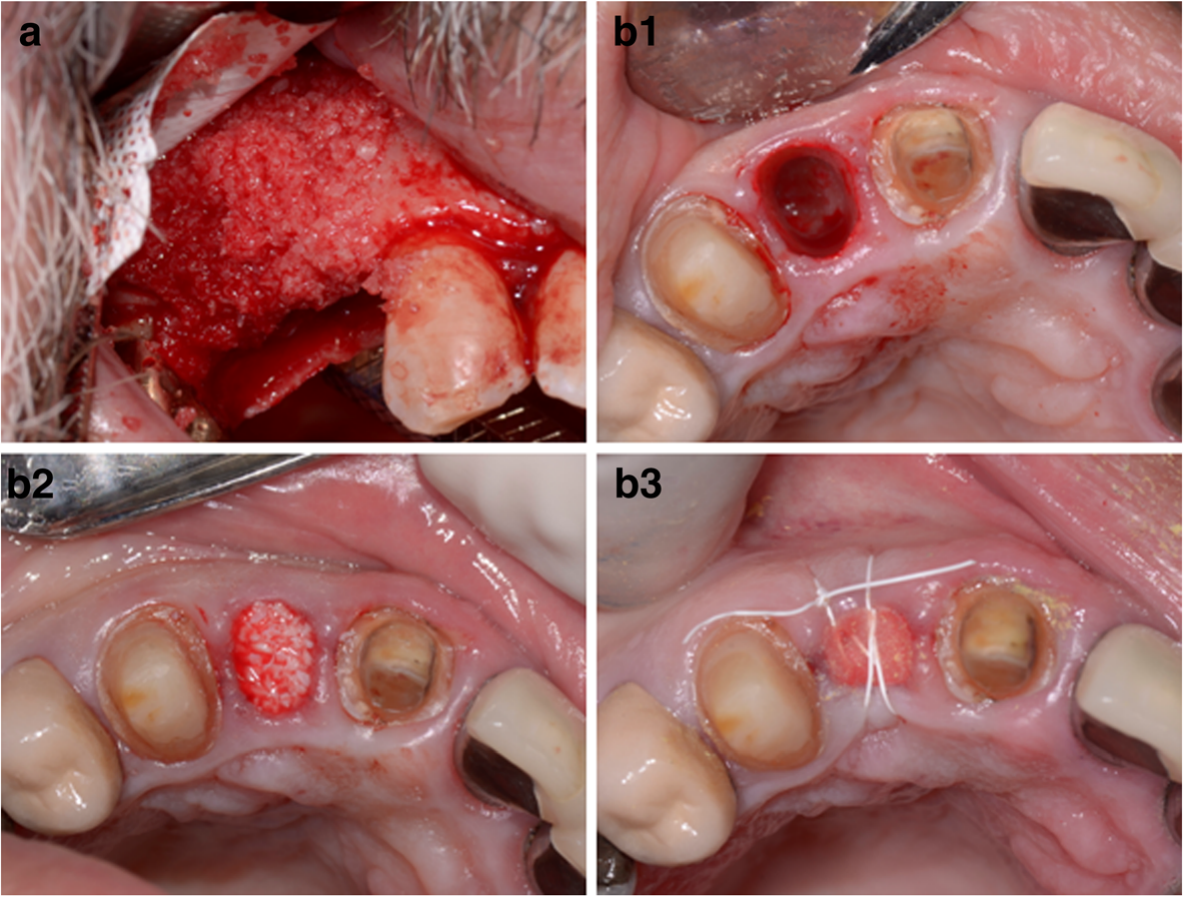
Clinical photographs showing two common applications of FDBA. **a**. Augmentation of resorbed alveolar ridge. **b**. Socket preservation after atraumatic extraction of teeth. **b1**. Tooth #1.2 was atraumatically extracted. **b2**. FDBA graft gently packed into extraction socket. **b3**. Absorbable collagen membrane used to cover bone graft

The freeze-drying to process these grafts for use distorts the 3D presentation of the human leukocyte antigens on surface of graft particles that affects the immune recognition [40, 41]. FDBA are known to be osteoconductive and can be combined with autografts to enhance the osteogenic potential [42, 43]. These graft tissues are mineralized and used for the treatment of periodontal defects [44–47]. Cortical FDBA demonstrate greater osteoinductive potential due to the growth factors stored in the matrix [48]. FDBA used in combination with absorbable barrier membranes have been used as replacement for autograft blocks for ridge augmentation [49]. The use of FDBA blocks for alveolar ridge grafting has shown presence of vital bone with a lamellar organization [50, 51]. Figure 2 shows two common application of FDBA.

#### Demineralized freeze-dried bone allografts (DFDBA)

These allografts which have been demineralized are used alone or in combination with FDBA and autografts. DFDBA undergo resorption at a quick rate [52, 53] and often have osteoinductive potential due to the bone morphogenetic proteins (BMPs) and growth factors present in the graft matrix [54]. DFDBA has been shown to produce less amount of vital new bone in comparison to autografts [55]. DFDBA acquired from younger cadavers have higher osteogenic potential in comparison with grafts from older individuals resulting in variation in BMP levels in different batches of DFDBA [56, 57].

### Xenografts

**Fig. 3:**
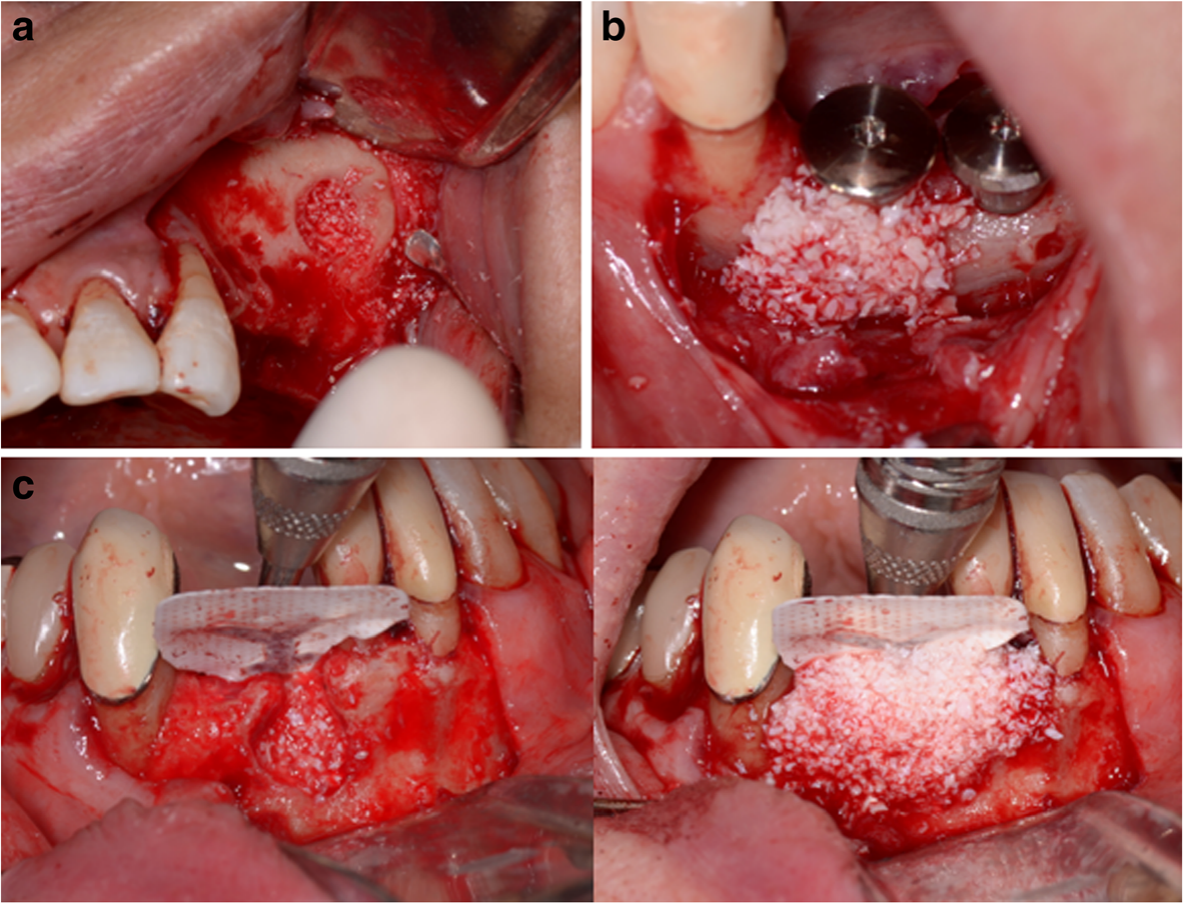
Examples of different applications of DBBM Xenograft (Bio-Oss®). **a** Subantral maxillary augmentation (direct sinus lift). **b** Augmenting thin bone around dental implants. **c** As a top layer covering FDBA particles to provide long-term support (sandwich GBR technique). Notice the use of Ti-reinforced d-PTFE membrane to provide space for the healing graft

Xenografts are graft tissues obtained from non-human species, i.e. animals and are usually osteoconductive with limited resorptive potential [58, 59]. The xenograft most commonly used in periodontal regeneration procedures is the deproteinized bovine bone mineral, commercially known as Bio-Oss®, which is a commercially available bone of bovine origin processed to yield natural bone mineral without the organic elements [60], (Fig. 3). After heat and chemical treatments, the inorganic phase of bovine bone consists mainly of hydroxyapatite (HA) that retains the porous architecture [61]. Although the heat and chemical processing removes most of the osteogenic components from bone, it does not completely eliminate the potential risk of disease transmission (bovine spongiform encephalopathy) and graft rejection but makes it a negligible possibility [62, 63]. Bovine-derived bone graft particles and blocks have been used for alveolar ridge augmentation procedures and intra-bony defect filling [64, 65].

Bio-Oss remains the most researched xenogeneic bone grafting material. Several research papers were published on the use of Bio-Oss in different surgical scenarios [66–70]. Of particular interest was the use of Bio-Oss as a graft material during direct sub-antral augmentation (sinus lift) procedures where dental implants placed in Bio-Oss grafts had survival rates at least similar if not better than autogenous grafts [71]. However, although bovine-derived bone block grafts have high osteoconductive potential, these grafts are inherently brittle and lack toughness. This makes them prone to failure during the screw fixation procedures and/or after implantation [65, 72].

In addition to bovine derived bone mineral, bone mineral can be obtained from other animal sources, such as equine or porcine source. Porcine bone graft tissue is a porous anorganic bone graft material consisting predominantly of calcium phosphate. These are supplied in granular form with a particle size of 0.25–1 mm and 1–2 mm (Gen-Os®) and are produced by removal of the organic components from porcine bone [73, 74]. The anorganic bone mineral matrix is biocompatible, having interconnecting macro- and microscopic porous structure that supports the formation and ingrowth of new bone at the implantation site [73]. A clinical study in humans in which porcine-derived graft was investigated for implant site development was showed to reduce the hard tissue resorption after tooth extraction [75].

The porous microstructure of marine coral has also been used as a template to fabricate porous coralline HA materials such as interpore-200® [76]. These materials are fabricated by coral being subjected to high temperature under pressurized treatment in the presence of aqueous phosphate solutions [77]. This converts the coral to calcium HA, while conserving the highly organized, permeable and interconnecting pore structure [76, 77]. These graft materials have an average pore diameter of 200 μm, and consists of about 60% porosity/void spaces [78, 79].

Calcium carbonate graft materials are of natural coralline origin and composed mostly of aragonite which is more than 98% calcium carbonate. Coralline calcium carbonate grafts have high osteoconductive potential allowing for new bone deposition to occur rapidly after implantation [80]. These grafts have a pore size of 100 to 200 μm, which is similar to that of cancellous bone. Also, they possess ~45% porosity that allows for greater resorption and new bone infiltration [30, 81]. These grafts have shown potential for improved defect filling in periodontal regeneration applications and do not undergo fibrous encapsulation [82–84].

### Alloplasts

Alloplastic synthetic biomaterials were developed to overcome the disadvantages of autografts and are fabricated in various forms with varying physicochemical properties and can be both degradable and non-degradable [14, 85–88]. Alloplasts are usually osteoconductive without any osteoinductive or osteogenic potential on their own and have been used extensively for periodontal regeneration [87]. The most routinely used alloplastic materials are HA, tricalcium phosphates (TCP) and bioactive glasses. Calcium phosphate biomaterials are of great interest to be used as bone replacement graft materials in periodontal regeneration as they have a similar composition to bone mineral, are osteoconductive, form bone apatite like material or carbonated HA and form a very strong bone-calcium phosphate biomaterial interface [14, 15].

#### Hydroxyapatite (HA)

This is a commonly used calcium phosphate biomaterial for bone regeneration applications due to having a composition and structure similar to natural bone mineral [89]. HA based grafts form a chemical bond directly to bone once implanted [90]. Synthetic HA is available and used in various forms: 1) Porous non-resorbable; 2) Solid non-resorbable; and 3) Resorbable (non-ceramic, porous) [91]. HA is non-osteogenic and mainly functions as an osteoconductive graft material. HA grafts show slow and limited resorptive potential and generally are dependent on passive dissolution in tissue fluid and cell mediated processes such as phagocytosis of particles for resorption [92, 93]. The degradation rate of HA depends on the method of ceramic formation, the calcium to phosphate ratio, crystallographic structure and porosity [92, 94]. The ability of HA to resorb is also heavily dependent upon the processing temperature. HA grafts synthesized at high temperatures are very dense with very limited biodegradibility [95]. These dense grafts are usually used as inert biocompatible fillers [96, 97]. At lower temperatures, the particulate HA is porous and undergoes slow resorption [98]. Early implant loading studies in alveolar ridges augmented with nano-structured HA has shown promise [99, 100]. Also, ridge augmentation with HA granules alone [100] or in combination with autografts has been investigated [101].

#### Tricalcium phosphate (TCP)

Over the last few years, TCP has been used and extensively investigated as a bone substitute. TCP has two crystallographic forms; α-TCP and β-TCP [102]. β-TCP exhibits good biocompatibility and osteoconductivity and is used commonly as a partially resorbable filler allowing replacement with newly formed bone [87]. Resorption of TCP grafts is thought to be dependent on dissolution by biological fluids in the absence of osteoclasts around the materials [103] and by presence of osteoclast mediated resorption based on the osteoclast-like giant cells in defect areas in many studies [104]. In terms of bone regenerative potential, β-TCP grafts have been shown to be similar to autogenous bone, FDBA, DFDBA and collagen sponge [105]. TCP biomaterials have been used in human clinical studies to repair periapical and marginal periodontal defects, as well as alveolar bony defects [106, 107]. In addition, there are studies using β-TCP that report alveolar ridge augmentation in vertical and horizontal dimensions with variable results [108–110].

#### Bioactive glass

These graft materials are composed of silicon dioxide, calcium oxide, sodium oxide, and phosphorus pentoxide [111, 112]. The particle sizes of bioactive glasses (Bio-Glass®) range from 90 to 710 μm to 300–355 μm [111, 113]. After implantation of bioactive glass, a silicon-rich gel is formed on the bioactive ceramic surface with the outer layer serving as a bonding surface for osteogenic cells and collagen fibers [114, 115]. Bioactive glass nanoparticles have been shown to induce cementoblasts to proliferate in an in vivo study [116]. Clinical reports of alveolar ridge grafting performed with bioactive glass reveal bone formation in close contact to the particles [111]. However, limited true periodontal regenerative outcomes based on human histological analysis has been demonstrated with the use of bioactive glass [117, 118].

#### Dicalcium phosphates (DCP)

These are acidic calcium phosphates that have a high solubility at physiological pH. Dicalcium phosphate dihydrate (DCPD or Brushite), has been investigated for both bone defect repair and vertical bone augmentation applications as injectable cements or as pre-set cement granules [119–121]. It has been demonstrated that injectable brushite cements are capable of regenerating bone in atrophic alveolar ridges, buccal dehiscence defects and maxillary sinus floor elevation procedures [122]. Bone growth in vertical direction obtained with brushite cement granules has been seen to be higher than that obtained with commercially available bovine HA materials [123]. However, brushite grafts after implantation undergo phase conversion to insoluble HA which ultimately limits their resorption rate and extent [102, 124].

Brushite can be used as precursor to the anhydrous form of DCP, dicalcium phosphate anhydrous, also known as DCPA or monetite. Monetite can be precipitated by dehydration of brushite or by modifying the precipitation conditions of brushite cements in order to favour DCP crystallization into monetite instead of brushite [102]. Monetite does not convert to HA after implantation [123–126] and resorbs at faster rates compared to brushite cement grafts [126–129]. Monetite granules have been compared with commercially available bovine HA (Bio-Oss®), and has shown greater resorption and bone formation in the extraction sockets [120].

#### Calcium polyphosphate (CPP)

Inorganic polyphosphates are polymers of orthophosphate, linked by energy-rich phosphoanhydride bonds to form polymeric chains. Calcium-Polyphosphate (CPP) is a good bone substitute as it can be made with mechanical properties similar to trabecular bone, controlled degradability and shows very good integration to host bone when implanted in vivo [130]. CPP has been used in different forms, such as sintered porous blocks [131], particulates [132] or nanoparticles [133]. Nelson et al. were the first to investigate CPP for bone regeneration as they explored its ability to repair canine mandibular alveolar defects. Assessment at 4 months showed increased bone and greater rates of union in the CPP group than in the bone graft control [134]. El Sayegh et al. demonstrated that the degradation rate of CPP did not substantially affect the interactions of human gingival fibroblasts with CPP materials but that compared with titanium alloy substrates, cell spreading and attachment were inhibited [135]. These studies suggest that CPP has promise as a biomaterial for biological and periodontal regenerative therapy [136].

#### Calcium sulphate

These compounds have a compressive strength greater than that of cancellous bone [137]. Calcium sulphate is usually applied as a barrier material to improve the clinical outcomes of periodontal regeneration therapy [138]. When used as a barrier, calcium sulphate materials work as an adjunct with other graft materials. A combination of β-TCP and calcium phosphate has been investigated which does not require a membrane, lowers cost, reduces surgical time, and has the potential to treat periodontal intrabony defects [139, 140]. A randomized controlled clinical trial over 12 months has shown that the use of calcium sulphate is useful in minimizing post-surgical recession when compared with the use of collagen membrane [139]. The clinical outcome of class II mandibular molar furcation defects has also been shown to be enhanced with the use of a mixture of calcium sulphate and DFDBA [141].

## Barrier membranes for periodontal guided regeneration applications

Periodontal regeneration by membrane techniques is based on the principal of separation of different tissues by surgical placement of physical barriers [142]. Soft tissue turnover rate is faster than bone and periodontal tissue formation, using barrier membranes allows for defect space to be maintained for regenerating tissues which would otherwise be infiltrated and occupied by the epithelial cells. If used in combination with bone grafts then the membranes also serve to stabilize, contain and preserve the graft materials [12]. This also results in reducing the rate of graft resorption [143, 144]. There are a variety of degradable and non-degradable barrier membranes that have been synthesized for periodontal GTR and GBR applications [11, 12]. The general characteristics that must be considered when designing barrier membranes intended for periodontal regeneration are: 1) biocompatibility; 2) cell-occlusivity; 3) Space-making ability; 4) Tissue integration; 5) Degradability; 6) Mechanical properties; and 7) Clinical handling characteristics [145, 146].

### Non-degradable barrier membranes

Materials such as cellulose acetate laboratory filters (Millipore®) [147–149]. silicone sheets [150] and expanded polytetrafluoroethylene (ePTFE) laboratory filters [146, 151], were the first non-degradable biomaterials used for investigating barrier membranes for regenerative therapy. Although these materials demonstrated some therapeutic potential, limitations such as inability to integrate with surrounding tissue, brittleness and the need to remove them after a certain period of time were observed [152, 153]. The function of non-degradable membranes is temporary as they maintain their structural integrity upon placement and are later retrieved via surgery. Although this gives the clinician greater control over the length of time the membrane will remain in place, the retrieval procedure increases the risk of surgical site morbidity and leaves the regenerated tissues susceptible to damage and post-surgery bacterial contamination [154]. Membrane exposure due to flap dehiscence during healing is also a frequent post-surgical complication [155]. However, in situations such as alveolar ridge augmentation prior to placement of dental implants, it may be desirable for the membrane to retain its functional characteristics long enough for adequate healing to occur, and then be removed. Hence, in specific situations, a non-degradable membrane provides more predictable performance [156, 157].

Barrier membranes used alone without particulate graft materials for guided regeneration applications are associated with membrane compression/collapse into the defect space by overlying soft tissue pressure [145]. To overcome this, membranes have been developed using stiff materials such as titanium membranes or metal reinforced expanded-polytetrafluoroethylene (ePTFE) [12] for the treatment of complex vertical periodontal defects [158]. In 1969, Boyne et al. first used a titanium mesh for the reconstruction of large osseous defects in edentulous maxillary ridges [159]. Titanium is a non-resorbable biomaterial and has been used extensively due to its high strength and rigidity and the resistance to corrosion [160, 161]. The rigidity of titanium provides excellent space maintenance and prevents collapse; and its plasticity permits bending and adaptation to any bony defect shape [162, 163]. Studies have shown that titanium mesh has been shown to maintain space predictably, even in cases with large bony defects [64, 164]. The commonly available and used titanium based mesh/membranes are the Frios®BoneShields, which is 0.1 mm thick and has a pore diameter of 0.03 mm [165, 166]; the Tocksystem MeshTM, which is 0.1–6.5 mm thick and a pore diameter of 0.1 mm and shows no sign of inflammation [165]; M-TAMTM which has excellent tissue compatibility and is 1700 μm thick and a pore diameter of 0.1-0.3 mm [167]; and the Ti-Micromesh ACE, which has a thickness of 1700 μm and 0.1 mm pore-size [168]. The common feature of the commercially available titanium membranes is the macroporosity which plays a critical role in maintaining blood supply and is thought to enhance regeneration by improving tissue integration and wound stability [169, 170]. However, this tissue integration can result in membrane removal difficult at the second surgery. Another problem associated with use of titanium membranes in guided regeneration therapy is the fibrous ingrowth and exposure of the membrane [171]. Development of less porous and micropore-sized titanium membranes could provide with improved clinical results.

**Fig. 4:**
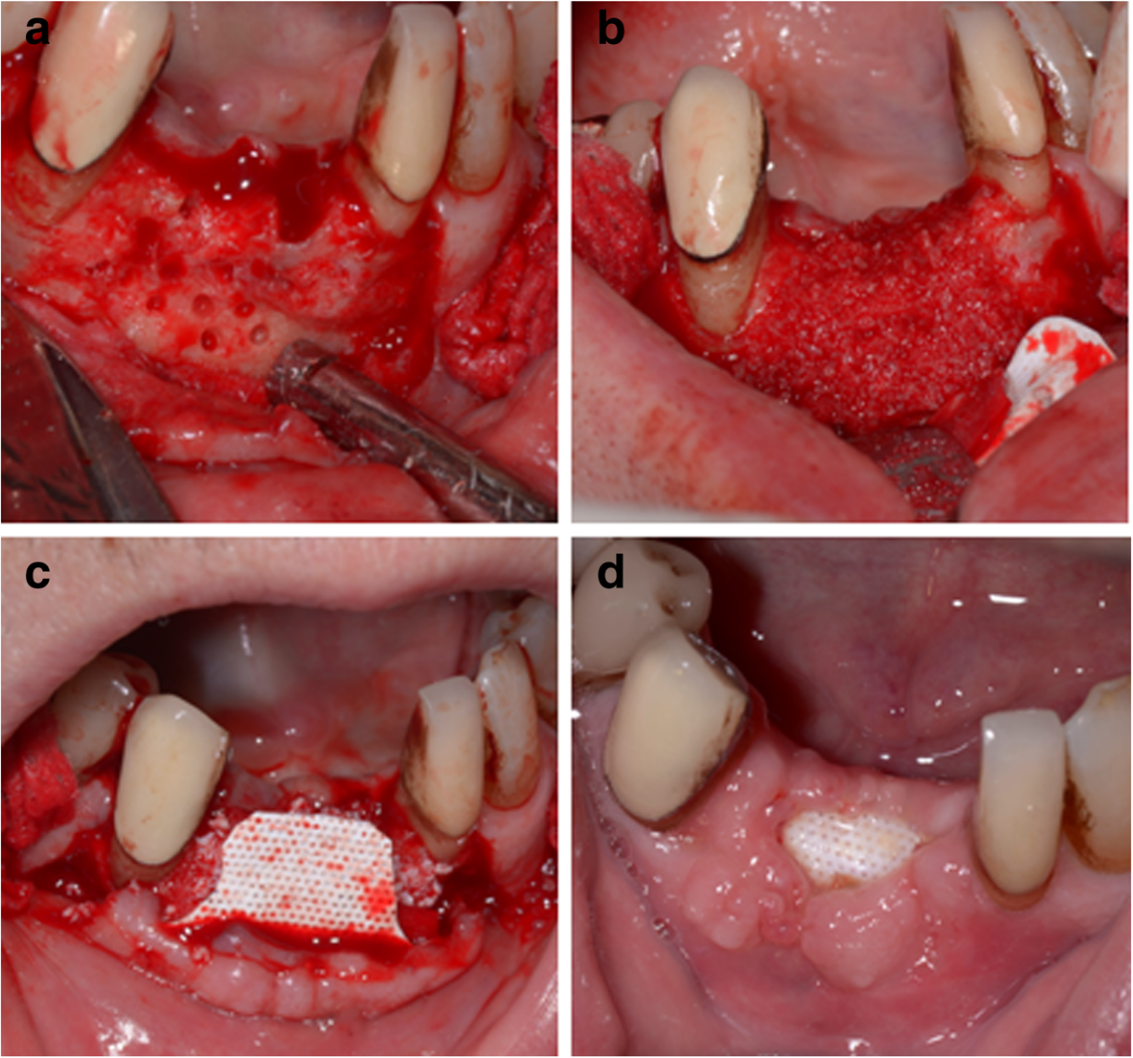
Clinical photographs showing Ti-reinforced d-PTFE membrane application. **a** Full-thickness mucoperiosteal flap reflected and one defect prepared to receive bone graft. **b** Particulate bone allograft (FDBA) gently packed into the bony defect. **c** Ti-reinforced d-PTFE membrane being adapted to cover the bone graft material. **d** Ti-reinforced d-PTFE membrane could be left exposed during the healing period thanks to its occlusive properties

Polytetrafluoroethylene (PTFE) is a non-porous inert and biocompatible fluorocarbon polymer [172]. Two non-resorbable PTFE based barrier membranes that are commonly used are the expanded-polytetrafluoroethylene (e-PTFE) and the titanium-reinforced high density polytetrafluoroethylene (Ti-d-PTFE). The e-PTFE has been commonly used in vascular surgeries [173] and is fabricated by exposing PTFE to high tensile stresses which results in expansion and the formation of a porous microstructure [174]. The e-PTFE membranes are stable in biological systems and their clinical effectiveness has been studied [175] with evidence of periodontal regeneration with their use [156]. When there is a clinical requirement that requires larger areas of space maintenance, Ti-d-PTFE can be used as it is stiffer due to the central portion of the membrane reinforced with titanium to prevent collapse [176]. The Ti-d-PTFE has also smaller pore size that does not allow bacterial ingrowth into the graft material if left exposed [177], (Fig. 4). An alternative approach is using a double layer of PTFE membrane with a titanium framework interposed (Cytoplast® Ti-250) which has shown to be successful for ridge augmentation and treatment of large defects in the alveolar process [178].

### Biodegradable barrier membranes

One of the major disadvantages of using non-degradable barrier membranes for periodontal regeneration application is that a second surgical procedure is required for removal. Hence, extensive research has been focused towards developing degradable barrier membranes. Clinical studies in the early 1990s reported the successful use of degradable membranes for GBR therapy [179–181]. Both natural and synthetic polymers have been investigated for this purpose with collagen and aliphatic polyesters being the mostly researched [182]. The main factors influencing safety and the effectiveness of degradable membranes are the degradation end-products and their fate. As the membrane degrades, particles or fragments are produced which may elicit a foreign body response. This results in a change in the biocompatibility profile of the membrane material [183] and can prevent bone formation and result in bone resorption [183, 184]. Therefore, it is important for the design of degradable membranes to be such that it maintains the functional characteristics for an adequate healing period. Currently, most commonly used degradable membranes are made of collagen or by polyglycolide and/or polylactide or copolymers of them [185]. The available biodegradable barrier membranes are mostly incapable in maintaining defect space on their own due to their lack of rigidity especially when exposed to oral fluids and/or blood. For this reason these membranes are frequently used in combination with autogenous or synthetic bone grafts substitutes [186, 187] with or without reinforcements, support screws and pins [188].

#### Natural degradable barrier membranes

Natural degradable barrier membranes are fabricated mostly using collagen from tissues from human or animal sources (Table 3). Collagen is used extensively in biomedical applications and can be acquired from animal intestines, skin and tendons [182]. Collagen has numerous biological properties which are desirable such as having low immunogenicity; attracting and activating gingival fibroblast cells and being haemostatic [189]. Collagen membranes have been shown to stimulate fibroblast DNA synthesis [180] and osteoblasts show improved adherence to collagen membrane surfaces in comparison to other barrier membrane surfaces [190]. The biodegradation of collagen membranes is accomplished by endogenous collagenases into carbon dioxide and water [189]. The degree of cross-linking of collagen fibers directly affects the rate of degradation with the relationship being inversely proportional [191].

**Table 3:** Common collagen based barrier membranes for clinical use [11, 189, 272]

Membrane	Constitution	Method of cross-linking	Tissue sources	Resorption time
BioGide	Types I & III collagen	None	Porcine (dermis)	24 weeks
BioMend	Type I collagen	Formaldehyde	Bovine (tendon)	6–8 weeks
BioMend-Extend	Type I collagen	Formaldehyde	Bovine (tendon)	18 weeks
Tissue Guide	Atelocollagen + tendon collagen	HMDIC^a^	Bovine (tendon + dermis)	4–8 weeks
BioBar	Type I collagen	N/A	Bovine (tendon)	24–32 weeks
Paroguide	Type I collagen (96%) & Chondroitin-4 sulfate (4%)	DPPA^b^	Calf skin	4–8 weeks
Biostite	Type I collagen (9.5%), Chondroitin-4 sulfate (2.5%) & HA^c^ (88%)	DPPA^b^	Calf skin	4–8 weeks
Periogen	Types I & III collagen	Gluteraldehyde	Bovine (dermis)	4–8 weeks
AlloDerm Regenerative Tissue Matrix (RTM)	Type I collagen	None	Human cadavers (skin)	28–36 weeks
Cytoplast RTM	Type I collagen	N/A	Bovine (tendon)	26–38 weeks

*HMDIC* ^a^ Hexamethylenediiscyanate

*DPPA* ^b^ Diphenylphosphorylazide

*HA* ^c^ Hydroxypatite

BioMend® is a biodegradable barrier membrane fabricated from Type-I collagen derived from bovine achilles tendon. The membrane is semi-occlusive, having a pore size 0.004 μm and resorbs in 4 to 8 weeks after implantation. Clinical results have revealed limited clinical effectiveness, highly dependent upon form and size of the defect [192]. To overcome the disadvantage of fast resorption, BioMend Extend® was later developed for use in cases that require the membrane to maintain its function longer than Biomend®. Biomend Extend® has an in vivo stability of around 18 weeks [193]. Bio-Gide® is a barrier membrane that resorbs in about 8 weeks and is synthesized from collagen Type-I and III derived from porcine skin source [194]. AlloDerm® Regenerative Tissue Matrix (RTM), is a collagen Type-I derived from human skin (Cadavers). The membrane thickness ranges from 0.9 to 1.6 mm and clinical applications include: root coverage, gingival augmentation, soft tissue ridge augmentation, and soft tissue augmentation around dental implants [195]. AlloDerm GBR® RTM is manufactured utilizing the same process used for AlloDerm® RTM and the membrane thickness ranges from 0.5 to 0.9 mm used for graft protection, containment and flap extension to achieve adequate primary closure [196]. Paroguide® is a collagen Type-I membrane enriched with chondroitin-sulphate. There have been reports of periodontal ligament regeneration and alveolar bone regeneration, with no signs of inflammation [152, 186]. Cytoplast RTM® i**s** synthesized with collagen Type-I derived from bovine tendon and is a multi-layered membrane which takes 26–38 weeks for complete resorption. It has an organized fiber orientation providing good handling and high tensile strength [197, 198].

**Fig. 5:**
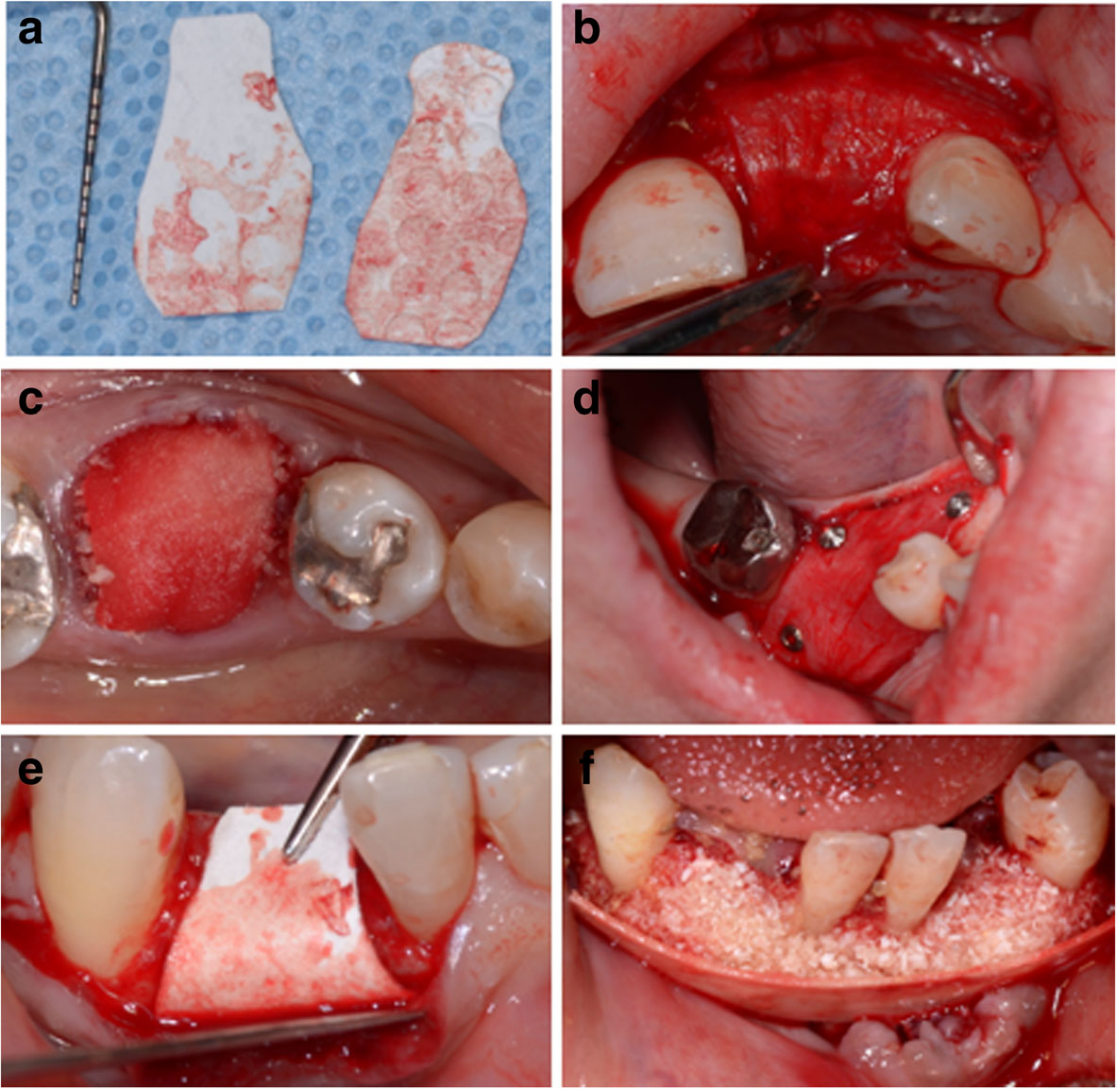
Degradable collagen membranes. **a** Collagen membranes are best cut into the desired shape utilizing a template before final insertion into oral cavity as their manipulation becomes more difficult after being mixed with blood. **b** Degradable porcine collagen membrane (Bio-Gide®) used to cover and contain FDBA particles during GBR. **c** Degradable porcine collagen membrane (Bio-Gide®) is often used to cover and contain FDBA particles used for socket preservation. **d** Degradable porcine collagen membrane (Bio-Gide®) can be stretched over bone graft and stabilized with fixation tacs. **e** For GBR, a degradable bovine collagen membrane (Biomend Extend®) could be chosen for its longer resorption time and stiffness **f** The stiff degradable bovine collagen membrane (Biomend Extend®) could be chosen for its relative rigidity and slow absorption time

A collagen membrane cross-Linked by diphenolphosphoryl azide is a Type-I collagen membrane, derived from calf pericardium has been investigated for regenerative applications. Although histology reveals significant inflammatory reaction [199], clinical studies have shown establishment of a connective tissue attachment is favored by the exclusion of the epithelium and gingival connective tissue during healing [152]. Collistat® is another collagen Type-I material which has demonstrated potential for GTR with the membrane completely resorbing seven days after implantation [200]. Chitosan is a polysaccharide comprising of copolymers of glucosamine and N-acetylglucosamine [201]. It has good biocompatibility and degradation appears to have no toxicity [202]. In addition it has bacteriostatic properties, the ability to inhibit growth of gram-negative and gram-positive bacteria, Actinobacillus actinomycetemcomitans and Streptococcus-mutans [203]. A chitosan based non-woven barrier membrane has been investigated that has a porous structure and is easy to manipulate [204]. It has shown the ability to form new bone and cementum in surgically created one-wall intrabony defects in beagle dogs [204]. Avitene® is a microfibrillar hemostatic collagen Type-I membrane derived from bovine corium. Histological evaluation after a clinical study has shown that this membrane was not clinically effective and is difficult to handle during surgery [205]. Figure 5 illustrates some clinical applications of two absorbable collagen membranes (Fig. 5).

#### Synthetic degradable barrier membranes

The most commonly used biomaterials used to fabricate synthetic degradable barrier membranes are the poly-α-hydroxy acids, which include polylactic polyglycolic acid and their copolymers [206]. The advantage of using polyhydroxy acids are that they undergo complete hydrolysis to water and carbon dioxide, which allows for complete removal from the implantation site [198]. However, the degradation rate varies depending on the presence glycols and lactides in the constitutional makeup [207]. Epi-Guide® is a porous three-layered and three-dimensional barrier membrane fabricated using polylactic acid polymers (D, D-L, L polylactic acid) and is completely resorbed in 6–12 months. The three-layered construction of the membrane attracts, traps, and retains fibroblasts and epithelial cells while maintaining space around the defect. Epi-Guide® is a self-supporting barrier membrane and can be used situations without support from bone grafting materials [186, 208]. Resolut LT® is a barrier membrane made of glycolide and lactic copolymer and a porous network of polyglycolide fiber that completely resorbs in about 5–6 months [172, 209].

Atrisorb® is barrier membrane that is prepared chair-side during the surgical procedure because it is made up of a polylactic polymer in a flowable form, dissolved in poly-dl-lactide and a solvent. It is composed of 37% of a liquid polymer of lactic acid that is dissolved in 63% N-methyl-2-pyrrolidone. This is flowed into a cassette containing 0.9% saline for ~5 min, after which the membrane having a thickness of 600–750 μm is obtained and cut to desired shape. The potential for periodontal regeneration has been investigated in both animal and human class II furcation defects where it demonstrated favorable regeneration [210]. Studies have reported its efficacy in the treatment of periodontal defects [211] and it resorbs completely in 6–12 months after implantation [212]. Treatment outcomes of GTR were investigated with using Atrisorb® in intrabony defects in a 3 year follow-up study [213]. The results showed that the outcome of treatment with Atrisorb® may be similar to open flap debridement [213]. A randomized controlled clinical trial showed that there was no regeneration when the biodegradable membrane Atrisorb® was used in combination with autogenous bone grafts [214].

Guidor® is a double-layered resorbable barrier membrane composed of both polylactic acid and a citric acid ester known as acetyl tributylcitrate. The external layer of the barrier membrane is designed with rectangular perforations allowing the integration of the overlying gingival flap. This surface design successfully promotes tissue integration and only limited gingival recession after usage has been reported [185, 215]. Between the internal and external layers, internal spacers are present that create space for tissue ingrowth. The internal layer has smaller circular perforations and outer spacers for maintaining the space between the membrane and the root surface. Studies have shown this membrane to be successful in the treatment of various periodontal defects [215]. Vicryl periodontal mesh® is made up of polyglactin 910 fibers which are copolymers of glycolide and L-lactide which form a tight woven mesh [216]. This barrier membrane has been shown to start resorbing after 2 weeks of implantation and completely resorbs in about 4 weeks [217]. Mempol® is manufactured from polydioxanon (PDS) with a bilayer structure. The first layer is covered with PDS loops 200 μm long to be used on the gingival side and is completely non-permeable [218, 219].

## Other strategies for periodontal regeneration

There is continuous research being conducted to develop newer strategies and technologies to achieve periodontal regeneration. Delivering modified genetic material (gene therapy) to periodontal cells to boost their regenerative potential by increasing the production and concentration of differentiation factors and growth factors is being investigated [220, 221]. A cellular tissue engineering approach has been investigated through which in vitro amplification of osteoblasts or osteoprogenitor cells grown on 3D constructs is carried out to increase the regenerative potential of bone [222–224]. Cell seeding of constructs with mesenchymal stem cells also has great potential to be used in the future [225, 226]. In addition, there has been great interest in using matrix derivatives (EMD), bone morphogenetic proteins (BMPs), platelet rich plasma (PRP) and exploring mineralization strategies for in situ attachment of periodontal membranes.

### Enamel Matrix Derivatives (EMD)

These are the purified fraction from the enamel layer of developing porcine teeth. It was assumed that those proteins, mostly made of amelogenins, might stimulate cementum deposition and periodontal regeneration [227]. A human histologic study reported that EMD can result in periodontal regeneration on previously periodontally diseased root surface. However, this finding was inconsistent [228]. Other studies reported that EMD with/ without the addition of a synthetic bone graft lead to clinical improvement in advanced intrabony defects [229, 230]. A recent review by the American Academy of Periodontology concluded that EMD is generally comparable with demineralized freeze-dried bone allograft and GTR in improving clinical parameters in the treatment of intrabony defects [231].

### Bone morphogenetic proteins (BMPs)

Through their chemotactic, mitogenic and differentiating mechanisms, BMPs play a crucial role in bone remodelling [232]. BMP use has shown promising results for intraoral applications such as sinus augmentation and alveolar ridge preservation [233–237]. The most commonly used and investigated BMPs for bone regeneration applications are BMP-2 & 7 [238]. The efficacy of BMP‑2, osteogenin, osteoprotein‑1 in an adult baboon model for regeneration in surgically created large furcation defects in the mandibular first and second molar has been investigated [239]. Also, significant periodontal regeneration of periodontal tissues was seen in periodontal defects treated with rhBMP-2 in beagle dogs [240]. It is worth mentioning that until recently BMP-2 has not been approved by the FDA for human intraoral applications as the carriers and dosage of BMP-2 and -7 were still under regulatory review and investigation. However, rhBMP-2 is now the only osteoinductive bone graft that has been tested and approved by the FDA as an alternative to autograft for sinus lift and alveolar ridge augmentation. In addition, rhBMP-2 has more Level 1 clinical evidence than any other bone grafting material [241].

BMP-2 may be more potent than BMP-7 as a bone forming agent due to its ability to induce both early and late osteogenic activity and matrix mineralization. BMP-7 assists primarily in later stages of bone formation. rhBMP-7 has not proven effectiveness and has therefore only received Humanitarian Device Exemption approval from the FDA [241]. It was also found that the addition of rhBMP-2 to augment post-extraction human buccal bone defects resulted in statically significant gain of bone when compared to a control. The bone available for the placement of a dental implant was approximately twice as great in the rhBMP-2, with an acellular collagen sponge as carrier, group compared to no treatment or placebo; with an increasing gradient based on increasing dosage of rhBMP-2 [242].

In general, despite those promising results, many clinician are still reporting minimal benefits if any of using BMPs and there is still some controversy that exists on the clinical effectiveness and safety of BMPs [243–245]. This might also be related to the improper use of the rhBMP that needs to stay in the region of repair to influence skeletal formation. For this to happen, the rhBMPs must be utilized with a suitable carrier such as a collagen sponge [246].

Further in depth studies are required for the development of delivery systems that can allow for controlled and precise release of BMPs for periodontal regeneration.

### Platelet-rich plasma (PRP)

PRP is an autogenous concentration of platelets in a small volume of plasma and is considered to be an extremely rich source of autogenous growth factors [247]. Separating PRP from patient blood and adding to bone graft materials is a new approach [248–250]. PRP has been used alone or in combination with autografts and allografts for the treatment of periodontal defects, extraction socket preservation, alveolar ridge augmentation, mandibular reconstruction, sinus floor elevation and maxillary cleft repair [251]. Results have shown greater volume and denser bone compared to autografts used alone for bone regeneration [252]. The improvement in the bone healing potential is believed to be due to the growth factors present in PRP [251], and several studies have reported positive results from PRP use on bone regeneration [253–257]. However, controversy still exists on PRP efficacy when used to treat infrabony periodontal defects. A recent meta-analysis on prospective clinical studies concluded that there was High heterogeneity among studies reporting on PRP in periodontal regeneration which made it difficult to draw clear conclusions. Nonetheless, within the limitations of that review, PRP might offer some beneficial effects on clinical and radiographic outcomes for regeneration of periodontal intrabony defects [258].

### Three-dimensional (3D) printing

The technology of three-dimensional (3D) printing allows for scaffold fabrication with high precision allowing for the creation of very detailed 3D structures [259]. Pathological or trauma induced damage to periodontal tissues can be potentially be treated by inducing bone-ligament complex regeneration using tissue engineered scaffolds [260, 261]. Direct 3D printing, stereolithography, selective laser sintering and fused deposition modeling are some of the common techniques used to fabricate scaffolds ranging from millimeter to nanometer size scale [262]. To achieve regeneration of complex tissue structures such as the periodontium, biomaterials are used as 3D templates for providing the extracellular matrix environment for the desired regenerative process [91]. Their efficacy of biomaterials for regenerating new periodontal ligaments has been shown in preclinical experiments [263]. A variety of natural and synthetic polymers can be used for 3D printing of scaffolds of desired configuration, size and architecture matching the defect [264, 265]. The use of faster resorbing polymers such as polylactic-coglycolic acid and gelatin as scaffolds with a highly porous structure has been shown to result in improved vascularization and tissue ingrowth [261, 266]. Further, 3D printed constructs cell based approaches and allow for the localized delivery biologics and osteogenic molecules such as bone morphogenetic proteins to potentially improve tissue growth, leading to more predictable periodontal regeneration. This can be achieved by the use of scaffolds that can provide biomechanical cues that allow for perpendicular alignment of periodontal fibers to the root surface, provide osteogenic cues and suitable space for bone regeneration and transport and stabilize cells capable of cementogenesis onto the root surface [260]. 3D printing strategy for achieving in vivo periodontal regeneration has great promise, however, there is a need for optimization and preclinical testing in large animal models and extensive human clinical trials to prove efficacy.

### Mineralization strategies for in situ attachment of periodontal membranes

The success or failure of periodontal regenerative procedures depends greatly on the structure of the surgical site, inter-individual variation and surgical skills. This variability is to a great degree due to the fact that none of the current membranes are designed to directly attach to the tooth surface and therefore provide a real barrier for migrating gingival epithelial cells. The result is epithelial down growth resulting in long junctional epithelium (JE), which is keratinized and provides no functional attachment [267, 268]. Since the mineralization of the basement membrane has been reported to mediate dentogingival adhesion in mammalian and non-mammalian vertebrates [269], exploring the feasibility of in situ mineralization can conceivably be used to create a physical attachment to a conditioned tooth surface. However, the applicability of this concept in vivo to provide functional attachment capable of inhibiting epithelial down growth remains to be demonstrated.

## The future of periodontal regeneration

A recent consensus report from the American Academy of Periodontology Regeneration Workshop reported that the application of protein and peptide therapy, cell-based therapy, genetic therapy, application of scaffolds, bone anabolics, and lasers were amongst the emerging technologies for periodontal regeneration and are expanding the potential of reconstructing the entire periodontal organ system. However, there is still insufficient evidence on emerging periodontal regenerative technologies to warrant definitive clinical recommendations [270]. In addition, several studies have demonstrated a good interaction between organic or inorganic scaffolds and adult stem cells in vitro. Thus, tissue engineering approaches have significantly and successfully enhanced the potential for bone regeneration in in vivo grafts. In the future, custom-made 3Dcomposite scaffolds grafted with stem cells and precisely tailored to complement the exact shape of the bone defect can be developed to facilitate complete restoration of defects in both hard and soft tissues [271].

## Conclusions

The development of biomaterials for periodontal regenerative applications is a challenge from engineering and a biological perspective. Extensive research has been carried out over the past few decades for the development of novel biomaterial options. Various hard tissue grafts and barrier membranes have been investigated for use in different combinations to promote periodontal regeneration. It is quite evident that the mechanical properties, biological behaviour and biodegradation mechanism vary for different graft materials. Dental surgeons need to be familiar with the clinical, biomaterials and biological factors involved in periodontal regeneration. With this, case selection, surgical technique, bone graft selection, membrane selection, and postoperative management can be directed towards obtaining the best clinical results. To date, there is no ideal biomaterial option or surgical technique that consistently provides perfect clinical results with regards to periodontal regeneration. Further extensive research is required with a need to focus on improving the biological interfacing between the graft material and the host tissues. Further approaches in the field of periodontal regeneration will rely on a combination of therapies with using improved biomaterial options. The future of periodontal repair/ regeneration seems promising with doors wide open for researchers to use new and emerging technologies in transforming predictable full periodontal regeneration from a being a dream into becoming a clinical reality.
